# Cationization of Cellulose Nanocrystals with Ionic Liquids and Polymers: From Electrostatic Screening to Adsorption-Controlled Aggregation

**DOI:** 10.1021/acs.langmuir.6c02450

**Published:** 2026-08-07

**Authors:** Vladimir Grachev, Viktória Hornok, Wim Thielemans, István Szilágyi

**Affiliations:** ‡ MTA-SZTE “Lendület” Biocolloids Research Group, Department of Physical Chemistry and Materials Science, Interdisciplinary Centre of Excellence, University of Szeged, 6720 Szeged, Hungary; § Sustainable Materials Lab, Department of Chemical Engineering, 97541 KU Leuven, Campus Kulak Kortrijk, 8500 Kortrijk, Belgium

## Abstract

Noncovalent surface modification of sulfated cellulose nanocrystals (sCNCs) and its effects on colloidal stability were investigated in the presence of alkylimidazolium ionic liquids (ILs) and cationic polymers. The addition of ILs resulted in the aggregation of sCNCs, and the exact mechanism of dispersion destabilization depended on the hydrophobicity of the ILs. Moderately hydrophobic ILs of short alkyl chains induced sCNC aggregation like inorganic salts. ILs bearing octyl or decyl groups adsorbed onto the sCNCs, leading to charge reversal of the particles, which was not sufficient to prevent aggregation. Adding water-soluble polycations in appropriate doses also reversed the charge of sCNCs upon surface adsorption without changing the morphology. The surface-coated particles maintained positive charge in the presence of salts and were stable at low ionic strengths, while ion-specific aggregation at higher salinity followed the trends predicted by the Hofmeister series of ions and Schulze–Hardy rule in mono- and multivalent salt solutions, respectively. The major interparticle forces and aggregation kinetics were rationalized within the DLVO model.

## Graphical abstract

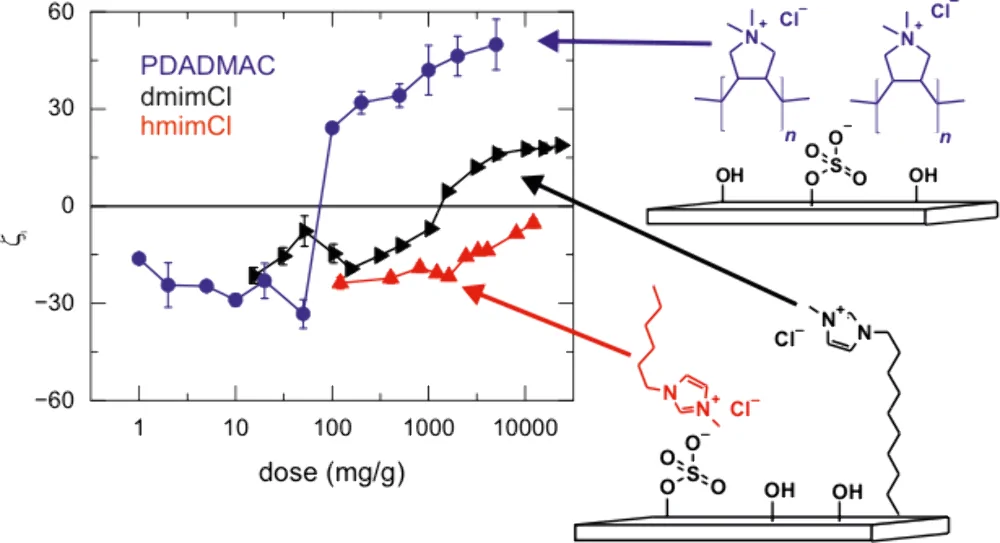

## 1 Introduction

Nanocellulose, and particularly cellulose nanocrystals (CNCs) produced through controlled hydrolysis of cellulose,^1–5^ has attracted a significant amount of contemporary interest in the scientific and industrial communities due to their intrinsic sustainability, large specific surface area, and low toxicity.^6^ CNCs obtained via cellulose hydrolysis are typically negatively charged owing to the presence of surface sulfate^7^ or phosphate functional groups or because their primary surface hydroxyl groups were oxidized to carboxylates,^8^ yet subsequent chemical modification also allows the production of hydrophobic and cationic CNCs. The latter, possessing a net positive surface charge, has attracted a significant amount of attention in a variety of possible applications, such as flocculants of colloidal suspensions^9,10^ and microalgae,^11^ Pickering emulsion stabilizers,^12^ drug delivery agents,^13^ templates for materials synthesis,^14^ flame retardants,^15^ surface modifiers,^16^ and structural components of injectable hydrogels.^17^ Moreover, they are promising platforms for the removal of emerging contaminants, including plastic particles and perfluorinated substances bearing negative charge.^18^

In these applications, cellulose nanomaterials are often dispersed in a liquid medium, highlighting the importance of the colloid and interface properties,^19–21^ which can be feasibly altered by surface modification, in both covalent and noncovalent ways. Conventional strategies used for cationization of CNCs often require the use of hazardous chemicals, such as pyridine^22^ and its derivatives,^23^ as well as glycidyltrimethylammonium chloride,^14^ for covalent modification, or toxic monomers such as *N,N*-dimethylacrylamide^24^ for the “grafting from” approach. Therefore, the development of facile and environmentally friendly methods for the preparation of positively charged CNC dispersions is a significantly important challenge.

Noncovalent cationization can, in principle, be achieved by adsorption of a surface-modifying agent onto CNCs, resulting in overcharging or charge reversal. These include cationic surfactants, e.g., cetyltrimethylammonium bromide (CTAB)^25^ or alkylammonium chlorides,^26^ and water-soluble cationic polymers, such as polydiallyldimethylammonium chloride (PDADMAC). Specifically, PDADMAC–nanocellulose complexes have found applications as templates for gold nanoparticle synthesis,^27^ dewatering agents,^28^ and interfacial emulsion stabilizers^29^ and in hydrogels for biomedical purposes,^30^ while polyethylenimine (PEI)-coated CNCs acted as nanocarriers in drug delivery processes.^31^ CTAB-modified CNCs were applied in coatings^32^ and as film-reinforcing components.^33^ Nevertheless, to achieve successful cationization, the resulting particles must possess sufficiently high dispersion stability once the surface modification is completed.

The colloidal stability of CNCs depends on surface chemistry and the composition of the surrounding medium. Charged CNCs usually form stable aqueous suspensions due to the electrostatic repulsion between the particles but lose stability at sufficiently high ionic strengths, where repulsion is suppressed, in agreement with the theory by Derjaguin, Landau, Verwey, and Overbeek (DLVO).^34,35^ This topic was extensively pursued in the presence of salts with different ionic valences^2,19^ and hydration features,^36^ as a function of pH,^20^ in surfactant solutions,^37^ and in hybrid CNC suspensions with other biopolymers.^21^ Nevertheless, today, little is known about the effect of larger cationic additives on the aggregative behavior of CNCs. The existing information is limited to the destabilization of CNCs in the presence of polyacrylamide^19^ and to the gelation of CNCs coated with cationic cellulose.^38^

Recently, ionic liquids (ILs) have shown good potential for optimization of dispersion stability due to their tunable structure and advantageous interfacial assemblies.^39^ In particular, sufficiently hydrophobic ILs enhanced the stability of various inorganic and organic nanoparticle suspensions through charge reversal.^40,41^ Hence, ILs can also be interesting candidates for noncovalent cationization of CNCs. Nevertheless, although ILs themselves have been widely used for cellulose processing^42^ and in nanocellulose analysis,^4^ the effective charge reversal of CNCs in the presence of ILs, as well as the effect of added ILs on the charge and colloidal properties of CNCs, has not yet been reported.

In this work, the charge and aggregation behavior of sulfated CNCs (sCNCs) were studied in the presence of cationic surface-modifying additives, such as alkylimidazolium-based ILs, surfactants, and polymers, with light scattering and microscopy techniques. The main goal was to develop stable dispersions of positively charged CNCs through noncovalent surface cationization, to assess the efficiency of cationization with different types of additives, and to reveal the governing interparticle forces in salt solutions.

## 2 Experimental Section

### 2.1 Materials

Sulfated cellulose nanocrystals (sCNCs) were obtained from CelluForce Inc., in the form of an aqueous suspension (concentration of 6 wt %). ILs, including 1-methylimidazolium chloride (mimCl, 98%), 1-ethyl-3-methylimidazolium chloride (emimCl, 98%), and 1-butyl-3-methylimidazolium chloride (bmimCl, 99%), were purchased from Iolitec GmbH. 1-Hexyl-3-methylimidazolium chloride (hmimCl, 97%), 1-methyl-3-octylimidazolium chloride (omimCl, 97%), 1-decyl-3-methylimidazolium chloride (dmimCl, 96%), poly­(diallyldimethylammonium chloride) (PDADMAC, 20% aqueous solution, average *M*_w_ = 200–350 kDa), and the polyacrylamide–PDADMAC copolymer (polyquaternium-7, PQ-7, 10% aqueous solution, acrylamide content of 55%, average *M*_w_ = 250 kDa) were acquired from Sigma-Aldrich. Sodium chloride (≥99%), sodium iodide (≥99.5%), sodium acetate trihydrate (99%), sodium bicarbonate (sodium hydrogen carbonate, 99.7%), trisodium citrate dihydrate (99%), calcium chloride dihydrate (99%), dodecyltrimethylammonium bromide (DTAB, ≥99%), and cetyltrimethylammonium bromide (CTAB, ≥99%) were purchased from VWR. Sodium bromide (99%) and branched polyethylenimine (bPEI, 30% aqueous solution, *M*_w_ = 70 kDa) were acquired from Alfa Aesar. Lanthanum chloride (anhydrous, 99.9%) was purchased from Fisher Scientific. Polyimidazolium chloride (IP-2) was synthesized at and obtained from EPFL (Switzerland).^43^ All products were used as received.

### 2.2 Atomic Force Microscopy

Atomic force microscopy (AFM) experiments were carried out on a Nanoscope IIIA Multimode system (Digital Instrument, USA), equipped with a TESP-SS-type antimony-doped silicon probe (Bruker, USA) (frequency of 230–410 kHz, *k* = 20–80 N/m, nominal tip radius = 2 nm, length = 110–140 μm, width = 25–35 μm). The diluted sCNC suspensions at a particle concentration of 100 ppm were deposited on freshly cleaned silicon substrates modified with poly­(allylamine hydrochloride) (PAH). Silicon substrates were submerged in a 0.01 wt % PAH solution for 1 min, subsequently immersed in a sCNC suspension for 1 min, rinsed with Milli-Q water, and dried using a nitrogen gas flow. Cellulose nanocrystals modified with IP-2, PDADMAC, and PQ-7 were deposited on silicon substrates without prior surface modification. Topographical imaging was performed in tapping mode.

Analysis of the length, width, and height of the particles was carried out using Gwyddion image analysis software^44^ on 5 μm × 5 μm images with at least 100 particles. The length (*L*) and width (*b*) were determined manually by measuring the distances determined from the size of the cross section in two perpendicular directions,^45^ while the height (*h*) of the individual particles was determined on background-corrected images. The distributions of the dimensions were fitted with a log-normal function in accordance with previous reports:^45,46^

1 $$y=\frac{A}{\sigma x}\;\exp\left(-\frac{1}{2}{\left(\frac{\ln\;x-\mu }{\sigma }\right)}^{2}\right)$$

where *A* is the normalization factor and μ and σ are the mean value and the standard deviation of the logarithm of measured variable *x*, respectively. The mean value ⟨*x*⟩ is calculated as

2 $$\left\langle x\right\rangle =\exp\left(\mu +\frac{\sigma^{2}}{2}\right)$$

where σ describes the width of the log-normal distribution and, hence, the polydispersity. The standard deviation of the measured variable Δ*x* is calculated as

3 $$\Delta x=\left\langle x\right\rangle \sqrt{\exp\left(\sigma^{2}\right)-1}$$

### 2.3 Elemental Analysis

The mass fractions of sulfur, hydrogen, carbon, and nitrogen in sCNCs were determined using a ThermoFisher Flash 2000 elemental analyzer for approximately 1 mg of lyophilized sCNCs. 2,5-Bis­(5-*tert*-butyl-2-benzo-oxazol-2-yl)­thiophene (BBOT) and enriched phenanthrene were used as calibration standards. Ten milligrams of vanadium pentoxide was added to the CNCs for an accurate determination of sulfur. The mass fractions were averaged over three separate measurements. The total sulfur content (*x*_S_) was calculated as

4 $$x_{S}=\frac{10\omega_{S}}{M_{S}}$$

where ω_S_ and *M*_S_ are the mass fraction of sulfur and the atomic mass of sulfur, respectively.

### 2.4 Dynamic Light Scattering

The dynamic light scattering (DLS) experiments were performed on a 3D LS spectrometer (LS Instruments, Switzerland), equipped with a fiber-coupled HeNe laser (638 nm wavelength and 120 mW maximal intensity). The hydrodynamic radii (*R*_h_) at a particle concentration of 5 mg/L were determined using the 3D cross-correlation technique. Correlation functions were collected at a scattering angle of 90° for 20 s with 100 runs performed for each time-resolved experiment and were fitted with the cumulant algorithm.

The apparent aggregation rate coefficient (*k*_aggr_) was obtained from the change in *R*_h_ over time:^47^

5 $$R_{h}\left(t\right)=R_{h,0}+k_{\mathrm{aggr}}t$$

where *R*_h,0_ is the apparent *R*_h_ of the individual particles at the onset of aggregation. The colloidal stability of the samples was quantified in terms of the stability ratio (*W*), a quantity widely used to describe the speed of particle aggregation in dispersions, which was calculated using the following equation:^40,48^

6 $$W=\frac{k_{\mathrm{aggr}\left(\mathrm{fast}\right)}}{k_{\mathrm{aggr}}}=\frac{{\left(\frac{dR_{h}\left(t\right)}{dt}\right)}_{t\to 0}^{\mathrm{fast}}}{{\left(\frac{dR_{h}\left(t\right)}{dt}\right)}_{t\to 0}}$$

where the index “fast” means diffusion-controlled aggregation of the particles. The value of *k*_aggr(fast)_ was determined for sCNCs in the presence of 1 M NaCl, under which conditions, the electrostatic repulsion is completely screened. The critical coagulation concentration (CCC) was calculated as

7 $$\mathrm{CCC}=10^{-a/b}$$

where parameters *a* and *b* are derived from the intercept and the slope of the log *W* plots at various concentrations of the additive:

8 log⁡W=a+blog⁡c

The distributions in *R*_h_ of bare and polymer-coated sCNCs were determined at a particle concentration of 5 mg/L in ultrapure water using at least 80 individual measurements and were fitted with a log-normal function similar to the AFM-derived dimensions.

In a typical sample preparation for DLS measurements, the stock sCNC suspension (6 wt %) was diluted to a concentration of 0.01 wt % (100 mg/L) with ultrapure water (Adrona), sonicated in an ultrasonic bath (Elmasonic, Germany) for 15 min, and filtered through a syringe filter with a pore size of 0.8 μm. Appropriate volumes of ultrapure water and solutions of additives (salts, ILs, surfactants, or cationic polymers, prefiltered through syringe filters with a pore size of 0.1 μm) were mixed in a glass cuvette. The sCNC suspension was then added, resulting in a particle concentration of 5 mg/L and a final sample volume of 2 mL. For the sample preparation of polymer-coated sCNCs, a stock dispersion was prepared by mixing an aliquot of the sCNC dispersion with a polymer solution at a desired dose (1 g/g for PDADMAC, IP-2, and bPEI and 5 g/g for PQ-7). The final concentration of the particle–polymer complex was 100 mg/L.

### 2.5 Electrophoretic Light Scattering

Electrophoretic light scattering (ELS) experiments were conducted on a LiteSizer 500 instrument (Anton Paar, Austria) equipped with a 40 mW semiconductor laser with a wavelength of 658 nm and operating in backscattering mode at a scattering angle of 175°. The measurements were performed on sCNC suspensions at a particle concentration of 5 mg/L. Samples were prepared using the same methodology as for DLS experiments. The electrophoretic mobilities (*u*) of the samples were measured using glass cuvettes with platinum electrodes (Univette, Anton Paar) with a final volume of 1 mL. The samples were homogenized on a vortex prior to the loading into the cuvette and equilibrated for 20 s in the device. The ζ potential was calculated using the Smoluchowski equation:^40^

9 $$\zeta =\frac{u\eta }{\varepsilon \varepsilon_{0}}$$

where η is the viscosity of the medium and ε and ε_0_ are the relative and the vacuum permittivity, respectively. The reported ζ potentials were averaged over at least five separate measurements.

## 3 Results and Discussion

### 3.1 Characterization of sCNCs

The AFM images of sCNCs and size distributions are displayed in Figure S1. The mean length, width, and height values were found to be 212 ± 52, 50 ± 10, and 5.5 ± 1.0 nm, respectively, and the polydispersity of each dimension was between 0.18 and 0.25. These results suggest that bare sCNCs have a ribbon-like shape with three distinct dimensions, similarly to previous reports.^45,49^ The effective hydrodynamic radius of bare sCNCs (*R*_h_) was found to be 45 ± 3 nm, as determined by DLS. It should be noted that for highly anisometric scattering objects such as sCNCs, *R*_h_ is a characteristic of Brownian motion of the particles in a liquid medium and equal to the radius of a sphere, which can be drawn around the particles and their hydration shell.^50^

The mass fraction of sulfur in bare sCNCs (Table S1) was found to be 5.96 ± 0.12 mg/g sCNCs. This corresponds to a total molar sulfur content of 186 ± 4 mmol/kg sCNCs, which is similar to the sulfur content in sulfated CNCs obtained by hydrolysis of cotton.^51^

### 3.2 Aggregation and Charge of sCNCs in the Presence of ILs and Surfactants

The colloidal behavior of sCNCs in the presence of alkylimidazolium ILs was evaluated by determining the *R*_h_ and the ζ potential data using time-resolved DLS and ELS, respectively. The results are shown in Figure 1.

**Figure 1:**
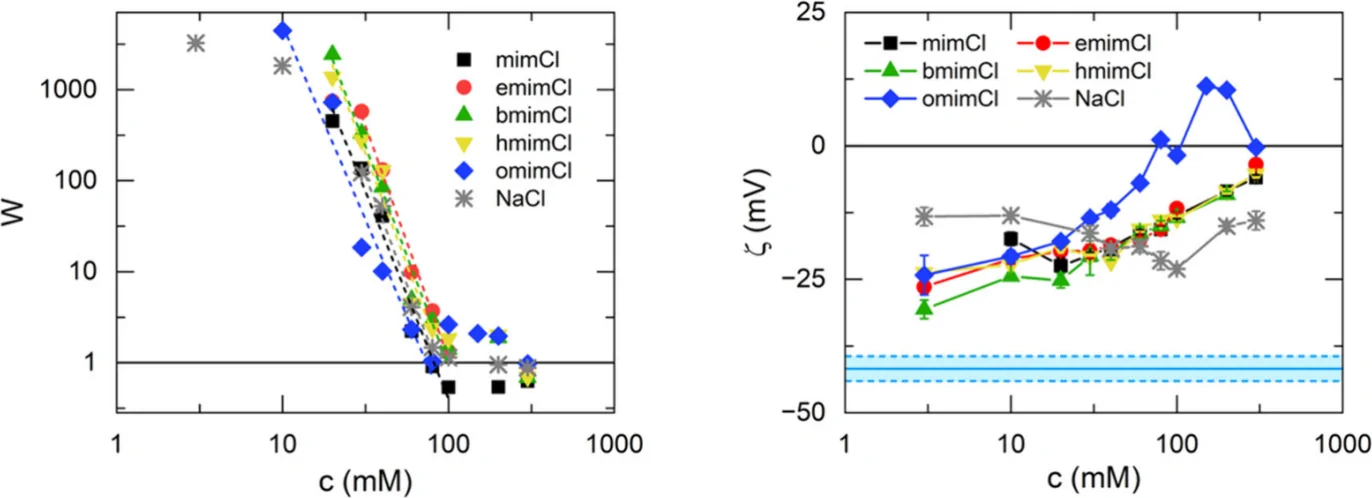
Stability ratio (*W*, left) and ζ potential (right) of sCNCs in the presence of alkylimidazolium ILs and NaCl at various concentrations. The light blue area represents the ζ potential value of sCNCs in pure water and corresponding standard deviations.

The addition of mimCl resulted in a gradual loss of stability of sCNCs, which is reflected in the decrease in *W*, whereas the magnitude of the ζ potential decreases, indicating that destabilization of sCNCs is caused by charge screening and subsequent loss of electrostatic repulsion between the particles.^34,35^ Similar destabilization was observed in the presence of more hydrophobic ILs, containing ethyl, butyl, hexyl, and octyl substituents. The calculated CCC data, displayed in Table S2, were found to be independent of the alkyl chain length, when the number of carbon atoms (*N*_C_) was less than 8. Moreover, the values were close to that measured in the presence of NaCl (Table S2) and KCl.^52^

The results of ELS indicated that surface overcharge of sCNCs took place in the presence of omimCl, while very similar ζ potential data were obtained for the other ILs with smaller *N*_C_ values. Such a charge reversal upon omim^+^ adsorption occurs at the isoelectric point (IEP (data in Table S2)), similar to polystyrene latex particles reported previously.^41^ In that latex–omimCl system, the formation of positive charge led to restabilization of the dispersions (i.e., stability ratios increased by a factor of 100) at high omimCl concentrations due to double-layer repulsion. Although a slight (about 3-fold) increase in the *W* data could be identified for sCNCs at larger IL doses, too, pronounced restabilization (i.e., more significant increase in *W* and, hence, more stable colloids) was prevented by the low magnitude of the ζ potential and, consequently, by the absence of strong electrical double-layer repulsion between the particles. The lack of sufficient electrostatic repulsion is also indicated by a decrease in the ζ potential to zero at the highest omimCl loading due to the charge screening effect of the chloride anions added to the samples at increased concentrations as part of the omimCl salt. An analogous phenomenon was also observed with other colloidal particles in the presence of ILs.^40^

In contrast to the present results, an increase in the affinity of ILs for clay surfaces from mimCl to hmimCl was observed previously.^40^ The CCC values also followed such a tendency with the highest value determined for mimCl and the lowest for hmimCl. Nevertheless, this was not obtained with the sCNCs. These findings imply that the sCNC particles are more hydrophilic, and hence, they are not sensitive to the moderate change in the hydrophobicity of the IL cations; however, the surface charge and aggregation are governed by the ionic strength determined by the concentration of ILs in these systems.

Further increasing the alkyl chain of ILs by the addition of dmimCl (*N*_C_ equal to 10) resulted in a large decrease in CCC, as shown in Figure 2. Moreover, the aggregation behavior of sCNCs in the presence of dmimCl (and, to a lesser extent, omimCl) was significantly different when compared to that of sCNCs with added ILs bearing shorter chains. At low concentrations of omimCl (10–20 mM) and dmimCl (1–3 mM), the *R*_h,0_ (the radius of the particles at the onset of the time-resolved DLS experiments) increased but remained constant thereafter, and hence, the particles were kinetically stable (Figure 2). It should be noted that the highest size values were measured close to the IL concentrations at the IEP (Table S2) due to the lack of electrostatic repulsion.

**Figure 2:**
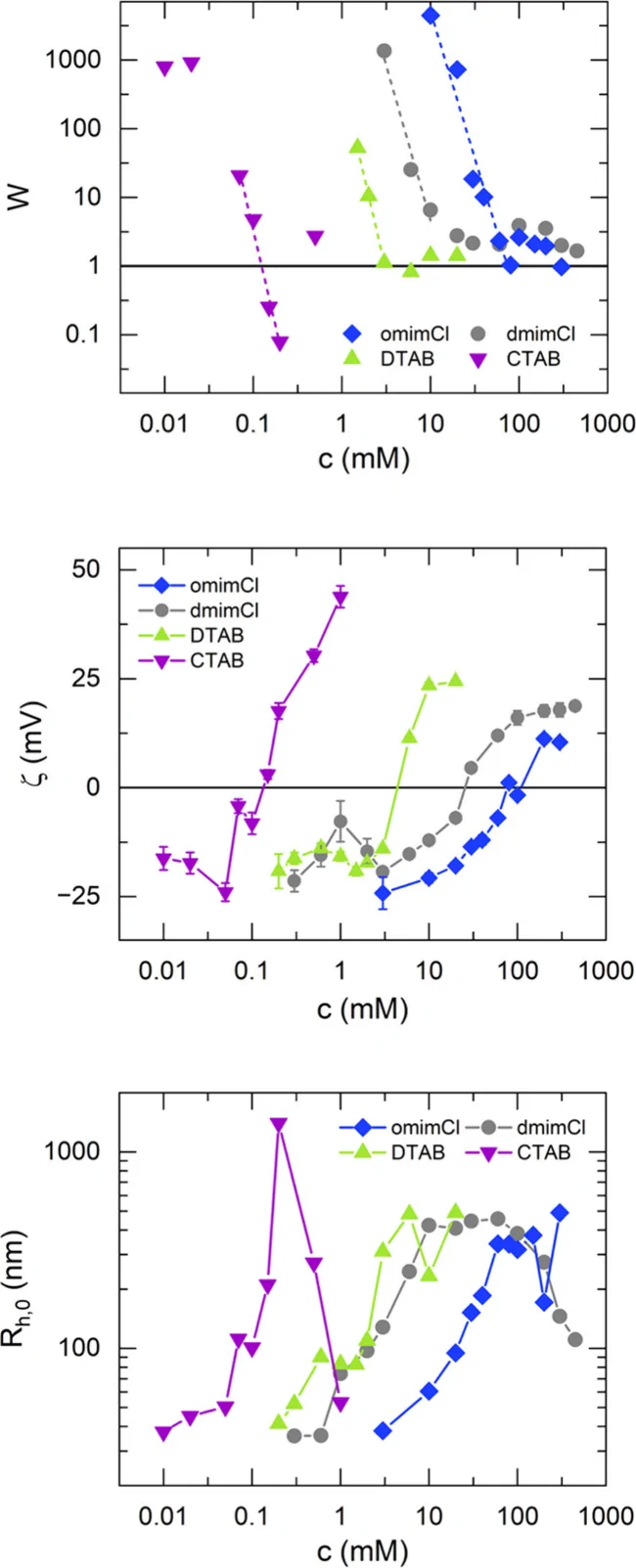
Stability ratio (*W*, top), ζ potential (center), and hydrodynamic radius at the onset of aggregation (*R*_h,0_, bottom) of sCNCs in the presence of hydrophobic ILs (omimCl and dmimCl) and cationic surfactants (DTAB and CTAB).

Similar behavior (i.e., charge reversal and aggregation at the IEP (see Figure 2)) of sCNCs was also observed in the presence of conventional cationic surfactants, namely, DTAB and CTAB, and the data were consistent with previously reported ones on CNC–surfactant interactions.^53^ The CCC values correspond to roughly 15–35% of the critical micelle concentration (cmc) of each surface-active molecule, as shown in Figure 3 and Table S2.

**Figure 3:**
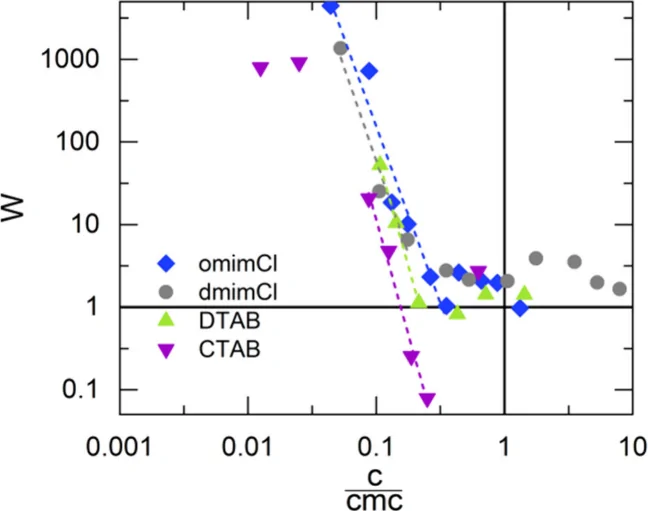
Stability ratio (*W*) of sCNCs in the presence of hydrophobic ILs and surfactants as a function of surfactant concentration (*c*) normalized to the cmc.

These data indicate that omimCl and dmimCl interact with sCNCs as cationic (i.e., oppositely charged) surfactants rather than hydrophobic salts. The increase in the hydrodynamic size of sCNCs at low surfactant concentrations could be explained by the formation of a surfactant monolayer on the surface of sCNCs with alkyl chains exposed to water.^53^ Moreover, surface aggregation of surfactants into bilayered structures or patches may occur, too.^54–56^ This gives rise to a moderate increase in the *R*_h_ of sCNCs but still provides stabilization for the particles. At higher concentrations of the surface-active substances (35–100% cmc), the aggregation of sCNCs takes place. In the CTAB system, sCNCs are stable and possess positive ζ potential, and their *R*_h_ is comparable to that of bare sCNCs at a concentration of 1 mM. These findings indicate that sCNCs became positively charged due to CTAB adsorption and the particles form stable dispersions under this experimental condition.

### 3.3 Surface Functionalization by Polymers

To further evaluate the noncovalent surface cationization of sCNCs, colloidal properties were investigated in the presence of cationic polymers (structures in Figure 4). First, a dose dependence of *R*_h_ and ζ potential was determined for each sCNC–polymer system over a broad range of concentrations. Then, aggregation and charge assessments were carried out on polymer-coated sCNCs in the presence of various mono-, di-, and trivalent ions to probe the effect of the different electrolytes. Such a comprehensive variation in the salt type and concentration was necessary to evaluate the mechanism of stabilization of the functionalized sCNCs, as well as their ion-specific sensitivity to electrolyte-induced aggregation.

**Figure 4:**
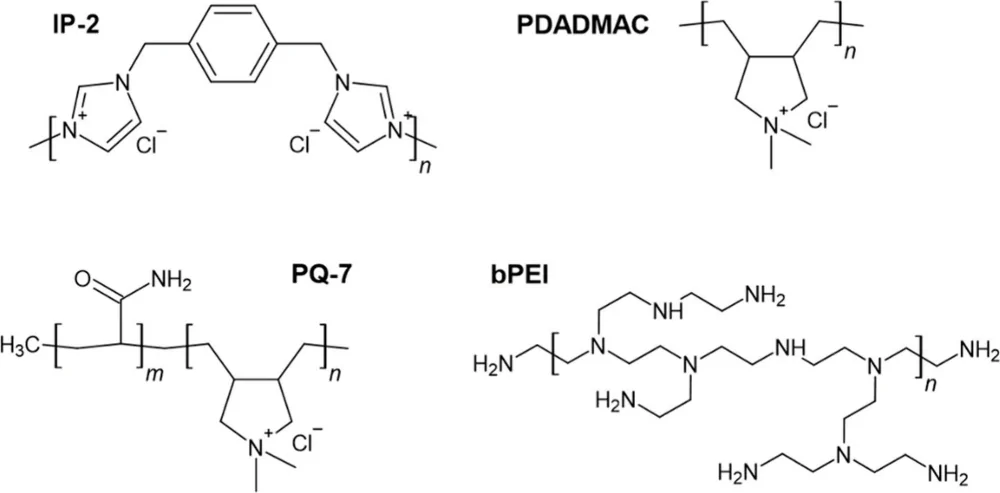
Chemical structures of cationic polymers used for surface cationization of sCNCs. Note that bPEI is presented in deprotonated form.

As shown in Figure 5, small doses of polycations (less than about 50 mg/g) did not alter the *R*_h_ or charge of sCNCs. At higher doses, *R*_h_ increased whereas ζ changed from negative to positive, indicating the surface overcharge. The sudden change in size at intermediate polymer concentrations can be qualitatively explained by DLVO theory. Accordingly, at the charge neutralization points, the electrical double layer vanishes, and thus, attractive dispersion forces constrain the particles to aggregate, giving rise to high *R*_h_ values. Therefore, the peak in the hydrodynamic size data is located near the IEP conditions. A similar phenomenon was reported earlier in other polycation–oppositely charged particle systems.^40,48,57^ Once the polymer dose was higher than 1 g/g, the *R*_h_ value remained constant while the ζ potential was positive, which indicates that sCNCs were coated, positively charged, and kinetically stable. Therefore, 1 g/g (equal polymer:sCNC mass ratio) was chosen as the dose of IP-2, PDADMAC, and bPEI for the future ion-specific studies in salt solutions. In the case of PQ-7, which is a copolymer consisting of cationic PDADMAC and neutral polyacrylamide units, the dose needed to stabilize the sCNCs was found to be 5 g/g.

**Figure 5:**
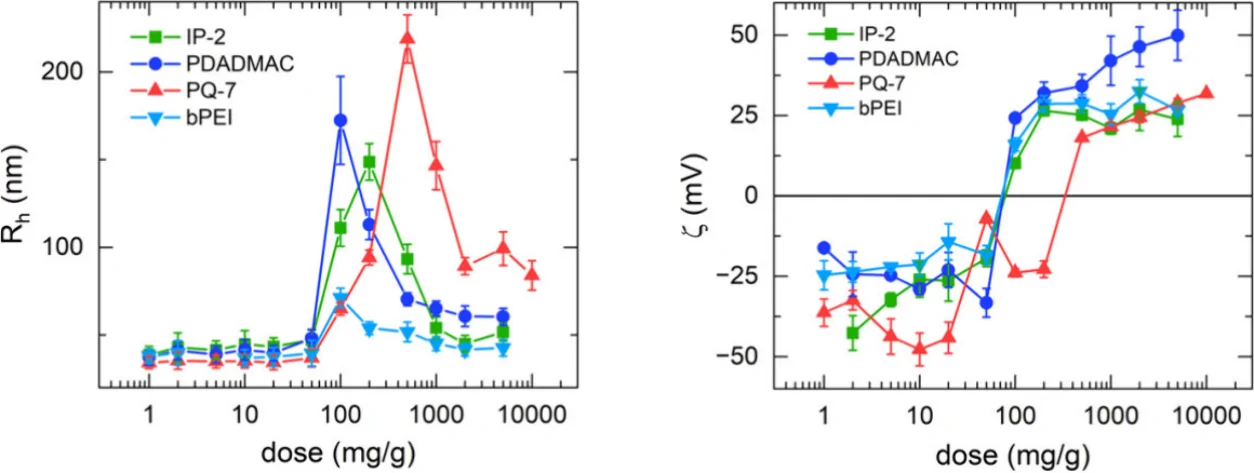
Hydrodynamic radius (*R*_h_, left) and ζ potential (right) of sCNCs as a function of cationic polymer dose (milligram of polymer per gram of sCNCs).

Coating sCNCs with a cationic polymer did not alter the morphology of the particles, as suggested by the AFM results (Figure S2 and Table S3). In addition, polymer-functionalized sCNCs exhibit smaller heights compared to those of the sCNCs, which could be explained by the fact that the former particles were deposited on the substrates directly, unlike bare sCNCs, which required the premodification of the substrates with PAH. Nevertheless, polymer-coated sCNCs had significantly larger *R*_h_ values compared to those of bare sCNCs, as shown in Figure S3. This finding is in line with previous results obtained for polymer-functionalized colloidal particles.^58^ Possible factors contributing to the increased *R*_h_ include the presence of kinetically stable aggregates or an increase in the volume of the particles upon coating, which cannot be observed in the AFM images.

### 3.4 Resistance against Salt-Induced Aggregation

The colloidal stability of polymer-coated sCNCs in the presence of salts was probed within time-resolved DLS and ELS experiments; i.e., sodium acetate, bicarbonate, chloride, bromide, and iodide were used to evaluate ion-specific aggregation, whereas sodium sulfate and citrate were used as salts containing di- and trivalent anions. Control measurements on bare sCNCs were performed in the presence of NaCl, CaCl_2_, and LaCl_3_ (Figure S4). In this way, the types and valences of the counterions of each particle were systematically varied in the experiments.

The dispersion properties of sCNCs coated with IP-2 were found to be sensitive to the addition of salts, as indicated in Figure 6. For different monovalent anions, the *W* and ζ potential data were very similar. The stability plots showed typical DLVO-type behavior. The decrease in the potentials with an increase in salt concentration is due to charge screening,^57^ whereas ion-specific effects can be hardly discovered within these series. Nevertheless, in the presence of multivalent anions, the charge and aggregation data are remarkably different from the monovalent case. Accordingly, aggregation took place at 10–100 times lower salt concentrations in the sulfate and citrate solutions in accordance with the Schulze–Hardy rule,^59,60^ which states an increase in the destabilization power if the valence of counterions increases. This could be explained by the higher affinity of multivalent ions for oppositely charged surfaces giving rise to weaker electrical double-layer forces, and thus, the particles aggregated due to the salts at lower concentrations. The different anion activities are reflected in the trends in the ζ potential data as they are shifted toward lower salt levels for multivalent ions (Figure 6). The values steeply decreased with the salt concentration; however, they remained positive over the entire range studied.

**Figure 6:**
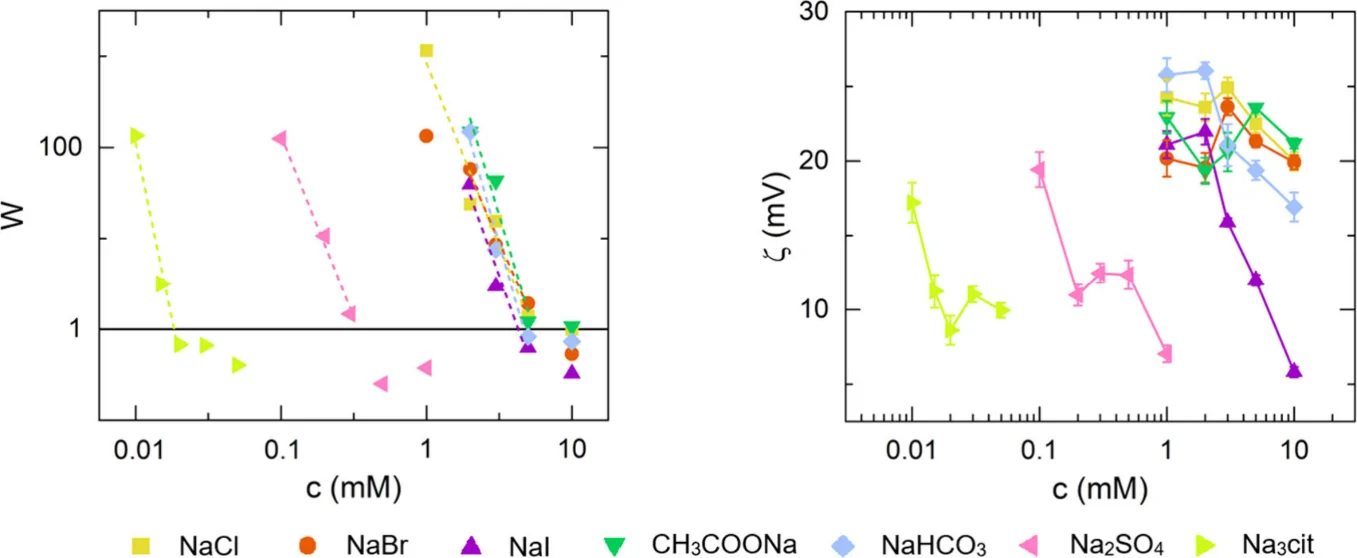
Stability ratio (*W*, left) and ζ potential (right) of IP-2-coated sCNCs in the presence of the sodium salt of various anions.

In general, PDADMAC-coated sCNCs aggregated at higher salt concentrations than IP-2-coated sCNCs, as shown in Figure 7. Aggregation of PDADMAC-coated sCNCs was found to be ion-specific even in monovalent salt solutions, which is reflected in a variation of the CCC values determined for different anions and apparent CCC tends to increase in the presence of more kosmotropic ions (CH_3_COO^–^ and HCO_3_^–^). In multivalent salt solutions, similar tendencies were obtained as in the case of sCNC-IP-2 systems, and the *W* data were shifted toward lower salt concentrations with an increase in the valence of the anions. The trend in the magnitude of ζ potential values is in line with the stability data. For instance, they are higher in the presence of kosmotropic ions suggesting higher CCCs. The potential values at the same concentration change in the same order as the CCC data, in both mono- and multivalent electrolytes.

**Figure 7:**
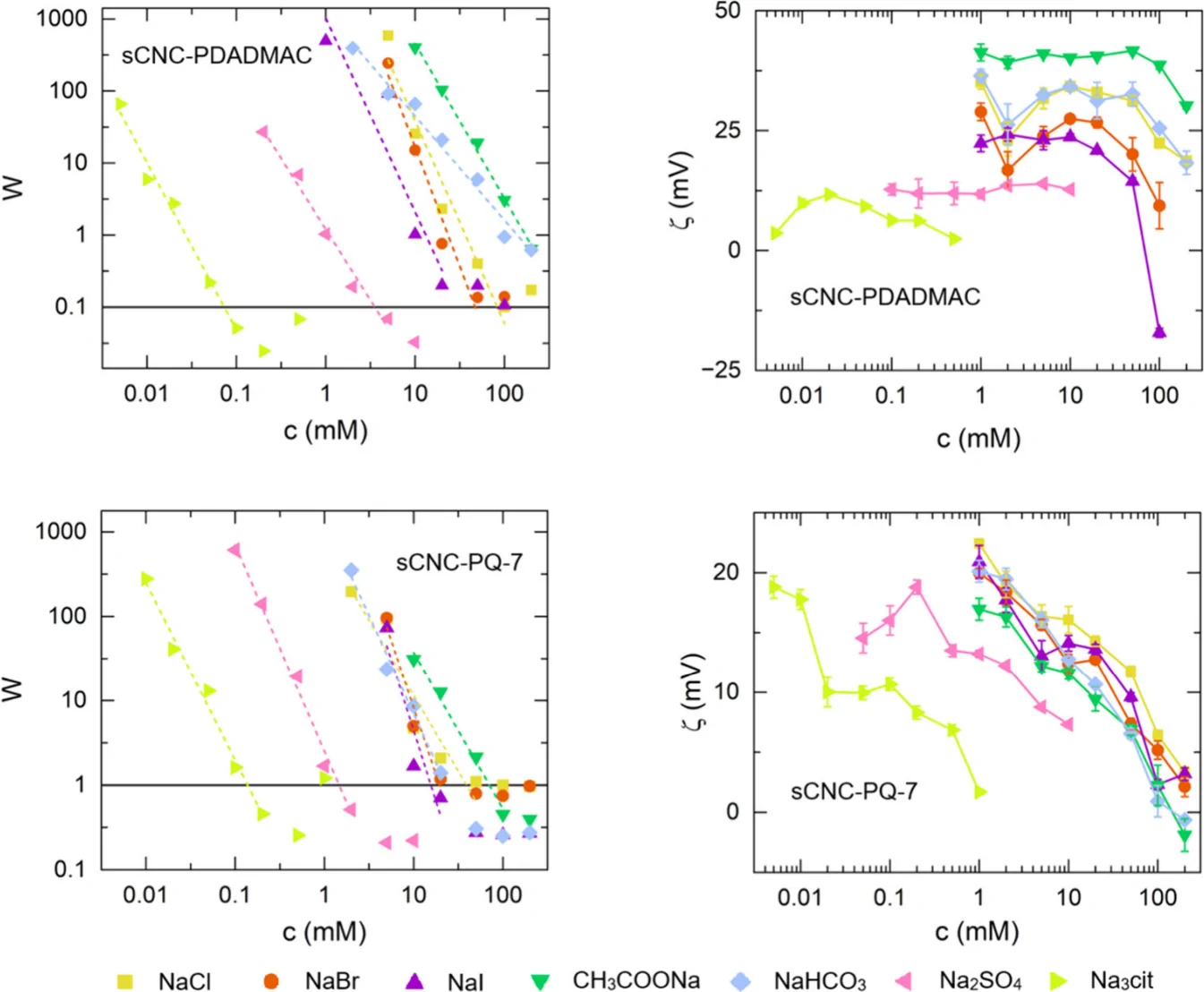
Stability ratio (*W*, left) and ζ potential (right) of PDADMAC-coated (top) and PQ-7-coated (bottom) sCNCs in the presence of various salts.

For PQ-7-coated sCNCs, ion specificity in aggregation is less pronounced than for the PDADMAC-functionalized ones, but the CCC data still tend to slightly decrease with an increase in the chaotropicity of the anion. The aggregation behavior of PQ-7-functionalized sCNCs was highly sensitive to multivalent ions, in accordance with Schulze–Hardy rule (detailed discussion below). Similar observations were taken for the ζ potential values, as they change significantly by varying the type of ions at the same concentrations and followed a similar tendency as the CCCs.

Regarding the system containing sCNCs and bPEI, no tests of colloidal stability in the presence of salts could be performed due to the appearance of visible flocs in the samples (Figure S5). This instability of bPEI-coated sCNCs is likely related to the particle concentration, since for the tests with salts, stock dispersions of polymer-coated CNCs were used at a loading of 100 mg/L (unlike 5 mg/L, which was used for all measurements for bare sCNCs, including the effect of polymer dose). At this higher concentration, sCNCs coated with other polymers did not form flocs, while the ζ potentials of bPEI-coated sCNCs at a polymer dose of 0.5 g/g were close to zero, giving rise to the appearance of aggregated clusters. Besides, at a polymer dose of 1 g/g, bPEI-coated sCNCs were positively charged but still remained in the flocculated state. This suggests that bPEI-induced flocculation of sCNCs is system-specific and, thus, may originate from the polymer conformation and protonation state on the surface. Another possible explanation can be the formation of Schiff bases between primary amine groups of bPEI and reducing end groups of cellulose.^61^ Previously, successful surface modification of sCNCs with bPEI was reported, while maintaining stability, but the polymer dose was smaller and, therefore, insufficient for surface charge reversal.^62^ Nevertheless, no experimental evidence could be obtained for the different tendency for flocculation in the case of the present bPEI-coated sCNCs.

To further evaluate the ion specificity during particle aggregation, the CCC values determined for polymer-coated sCNCs in the presence of monovalent salts are presented in Figure 8 and listed in Table S4. The PDADMAC-coated sCNCs exhibit the highest stability among all systems indicated by the highest CCC data in each electrolyte solution.

**Figure 8:**
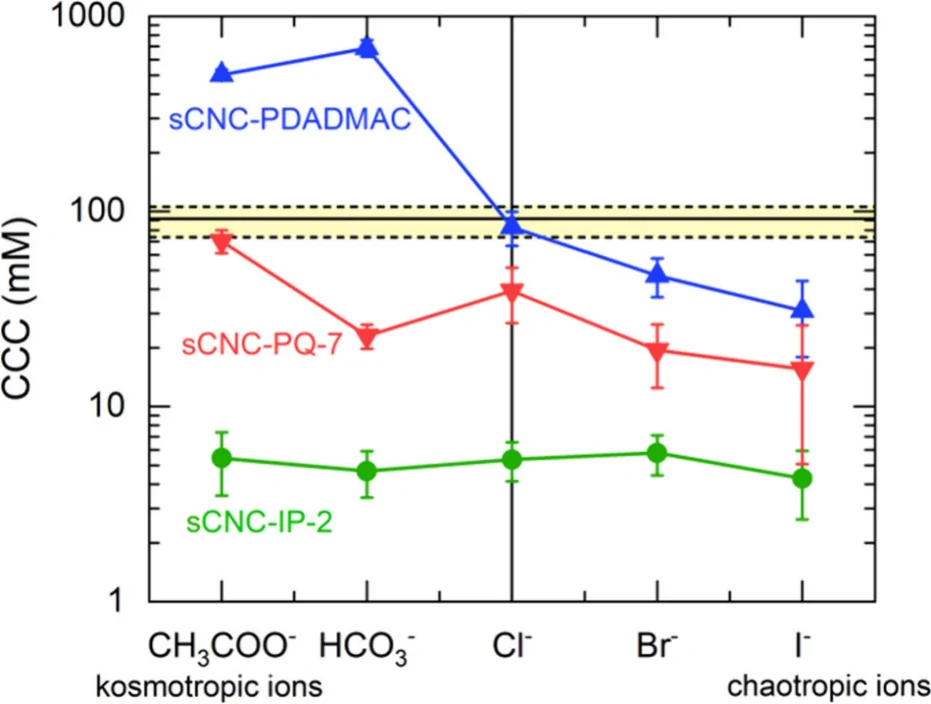
Critical coagulation concentration (CCC) data of polycation-coated sCNCs in the presence of various monovalent counterions. The yellow (between dashed lines) area indicates the CCC (with error range) of bare sCNCs measured in a NaCl solution.

The trend in the CCC data measured for the sCNC–PDADMAC system shows pronounced ion specificity, which aligns well with the classical Hofmeister series of ions^63^ also reported for destabilization of particle dispersions by salts.^36,41,60^ Accordingly, kosmotropic anions like acetate prefer to remain in a hydrated state in the bulk solutions, while chaotropic ones such as iodide favor adsorption on the oppositely charged surface. The latter scenario leads to a decrease in the particle charge and weaker electrical double-layer forces. Subsequently, a lower concentration of the salt is needed to reach the CCC, i.e., to induce aggregation by van der Waals forces if only DLVO-type interactions are present. The CCC values determined with the other halide ions are also in line with the prediction of Hofmeister theory. Such a tendency can be observed in the sCNC–PQ-7 and sCNC–IP-2 systems, too; nevertheless, the dependence on the type of anions is less pronounced in these cases, most likely due to extensive ion condensation into the surface polymer layer.^64^

Moreover, the CCC data measured for polymer-coated particles in multivalent salt solutions decreased with the valence of the counterions (Figure 9 and Table S5) in agreement with the Schulze–Hardy rule.^59^

**Figure 9:**
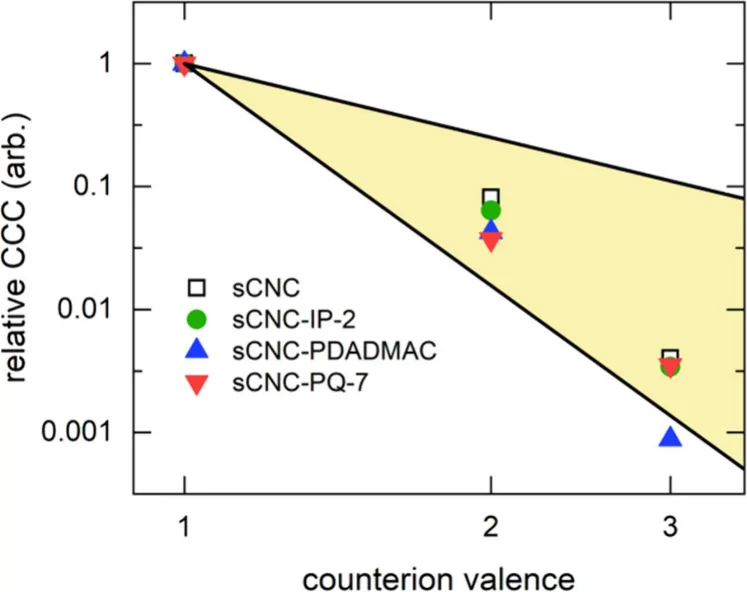
Relative CCC of polymer-coated sCNCs as a function of counterion valence. The yellow area represents the validity range of the Schulze–Hardy rule; black straight lines indicate upper and lower validity limits of the Schulze–Hardy rule.

This law states a CCC ∼ *z* ^–*x*^ relation, where *z* is the counterion valence and *x* is 1.6 and 6.5 for weakly and strongly charged particles, respectively. Its validity has been already proven for charged colloidal particles dispersed in various salt solutions.^36,40,60^ In the present system, the relative CCC data fell within the above limits of exponent *x*, while the sCNC–PDADMAC system showed a slightly stronger dependence in the case of the trivalent ions. However, these results also confirm the presence of DLVO-type interparticle forces responsible for colloidal stability, as the Schulze–Hardy rule can be directly derived from this theory.^59^

The above results clearly indicate that specific effects of mono- and multivalent ions on particle charge and aggregation are present to different extents depending on the type of polymers used for surface functionalization. This information implies that such effects on the colloidal stability of sCNCs can be optimized by applying appropriate coating agents and considering the electrolyte content of the dispersions.

## 4 Conclusions

The colloidal behavior of sCNCs was altered by the addition of various positively charged surface-modifying agents to cationize the particles. On the basis of their effect on the charge and stability of sCNCs, these additives can be classified into the following groups. First, hydrophobic salts, which include alkylimidazolium ILs with alkyl chains of short and moderate lengths, destabilize sCNCs similarly to simple inorganic salts and do not cause surface overcharge. Second, ionic surfactants, including alkylimidazolium ILs with long alkyl chains and conventional cationic compounds (DTAB and CTAB), show higher affinity for the surface of sCNCs, leading to overcharging at the IEP, an increase in *R*_h_, and destabilization of the dispersions already at low concentrations. Third, polycations, such as IP-2, PDADMAC, and PQ-7, strongly adsorb onto sCNCs, forming kinetically stable and positively charged particles without altering their morphology. The polymer-coated sCNCs maintained a positive charge upon addition of salts with destabilization effects described by the Hofmeister series for monovalent counterions and the Schulze–Hardy rule for multivalent ones. The ion-specific behavior of the functionalized sCNCs was found to be dependent on the chemical composition of the applied polymer. These findings revealed that polycation-coated sCNCs could be a more sustainable alternative to the covalently modified or grafted cationic sCNCs, owing to their persistent positive charge and sufficient colloidal stability for further use. It should be noted that the concentration range of sCNCs used in this work was limited to 0.01 wt % (100 mg/L), which necessitates further study of colloidal properties of polymer-coated sCNCs at higher concentrations. The insights provide rational design guidelines for tailoring charge-controlled and ion-responsive nanocellulose dispersions in water treatment, colloidal formulations, and advanced biobased material systems operating under variable salinity conditions.

## Supplementary Material



## Notes

AFM images of sCNCs, size distribution, CCC, stability ratio, elemental analysis, and ζ potential data (PDF)

## References

[1] D’Acierno F., Duchemin B., Capron I. (2026). Temperature-dependent underoil wettability
of cellulose nanocrystals and their biobased modifications. Langmuir.

[2] Ballu K., Lim J. H., Parton T. G., Parker R. M., Frka-Petesic B., Lapkin A. A., Ogawa Y., Vignolini S. (2025). Tailoring
the morphology of cellulose nanocrystals via controlled aggregation. ACS Nano.

[3] Cao G., Xu J., Han L., Wang Y., Zhao W., Zhou X., Lee Y., Loh W., Tam K. C. (2025). Interactions between cellulose nanocrystals
and conventional/gemini surfactants. Carbohydr.
Polym..

[4] Fliri L., Heise K., Koso T., Todorov A. R., Del Cerro D. R., Hietala S., Fiskari J., Kilpelainen I., Hummel M., King A. W. T. (2023). Solution-state
nuclear magnetic resonance
spectroscopy of crystalline cellulosic materials using a direct dissolution
ionic liquid electrolyte. Nat. Protoc..

[5] Rodrigues P. D., Myrdek T., Ferreira G. A. (2026). Foam stability
mediated by cellulose
nanocrystal-anionic surfactant interactions. Langmuir.

[6] Qiu J. M., Lu C. H., Xiong R. (2026). Bioinspired nanofluidic-assisted
printing cellulose nanocrystal photonic patterns. ACS Nano.

[7] Beck-Candanedo S., Roman M., Gray D. G. (2005). Effect of reaction conditions on
the properties and behavior of wood cellulose nanocrystal suspensions. Biomacromolecules.

[8] Montanari S., Roumani M., Heux L., Vignon M. R. (2005). Topochemistry of
carboxylated cellulose nanocrystals resulting from TEMPO-mediated
oxidation. Macromolecules.

[9] Haleem N., Zhang C., Jamal Y., Albert K., Fan D., Yao B., Hussain F., Yang X. (2023). Preparation of cationic cellulose
as a natural flocculant/sorbent and its application in three water
treatment scenarios. Water.

[10] Blockx J., Verfaillie A., Deschaume O., Bartic C., Muylaert K., Thielemans W. (2021). Glycine betaine grafted nanocellulose as an effective
and bio-based cationic nanocellulose flocculant for wastewater treatment
and microalgal harvesting. Nanoscale Adv..

[11] Pires
Maciel A., Thielemans W., Muylaert K., Formosa-Dague C. (2025). Investigating
the nanoscale dynamics of chlorella vulgaris flocculation with pyridinium-modified
cellulose nanocrystals. Langmuir.

[12] Guo S., Zhu Y., Xu W., Huan S., Li J., Song T., Bai L., Rojas O. J. (2023). Heteroaggregation effects on Pickering stabilization
using oppositely charged cellulose nanocrystal and nanochitin. Carbohydr. Polym..

[13] Lin N., Geze A., Wouessidjewe D., Huang J., Dufresne A. (2016). Biocompatible
double-membrane hydrogels from cationic cellulose nanocrystals and
anionic alginate as complexing drugs codelivery. ACS Appl. Mater. Interfaces.

[14] Zou R., Lyu L., Chansai S., Hurd J., Xin R., Wong J. A. C., Lee D., Hardacre C., Jiao Y., Fan X., Ou X. (2025). Cationic cellulose nanocrystals assisted synthesis of mesoporous
Silicalite-1 zeolites with fewer silanol defects. Microporous Mesoporous Mater..

[15] Long G., Ma X., Yu H. Y., Jin M. J., Chen X. F., Wang X. H., Yao Z. W., Ge D. T., Zeng C., Wang S. J., Zang H. J., Jiang Y. (2026). Zn2+-DTPMPA chelated cellulose nanocrystal
hybrid: synergistic flame-retardant, antibacterial and mechanical
reinforcement mechanisms for PA66 composites. Carbohydr. Polym..

[16] Cao G., Zhao W., Han L., Teng Y., Xu S., Nguyen H., Tam K. C. (2025). Enhancing
droplet spreading on a
hydrophobic plant surface by surfactant/cellulose nanocrystal complexes. ACS Nano.

[17] Warwar
Damouny C., Martin P., Vasilyev G., Vilensky R., Fadul R., Redenski I., Srouji S., Zussman E. (2022). Injectable
hydrogels based on inter-polyelectrolyte interactions between hyaluronic
acid, gelatin, and cationic cellulose nanocrystals. Biomacromolecules.

[18] Franco L. A., Stuart T. D., Hossain M. S., Ramarao B. V., VanLeuven C. C. W., Wriedt M., Satchwell M., Kumar D. (2024). Apple pomace-derived
cationic cellulose nanocrystals for PFAS removal from contaminated
water. Processes.

[19] Zhong L., Fu S., Peng X., Zhan H., Sun R. (2012). Colloidal stability
of negatively charged cellulose nanocrystalline in aqueous systems. Carbohydr. Polym..

[20] Antoniw J. M., Hallman M. T., Kiriakou M. V., Morse T., Cranston E. D. (2023). Colloidal
stability window for carboxylated cellulose nanocrystals: considerations
for handling, characterization, and formulation. Langmuir.

[21] Bose A., Zakani B., Grecov D. (2025). Influence of buffer on colloidal
stability, microstructure, and rheology of cellulose nanocrystals
in hyaluronic acid suspensions. J. Colloid Interface
Sci..

[22] Jasmani L., Eyley S., Schütz C., Van Gorp H., De Feyter S., Thielemans W. (2016). One-pot functionalization of cellulose nanocrystals
with various cationic groups. Cellulose.

[23] Imtiaz Y., Tuga B., Smith C. W., Rabideau A., Nguyen L., Liu Y., Hrapovic S., Ckless K., Sunasee R. (2020). Synthesis and cytotoxicity
studies of wood-based cationic cellulose nanocrystals as potential
immunomodulators. Nanomaterials.

[24] Liu T., Ding E., Xue F. (2017). Polyacrylamide
and poly­(N,N-dimethylacrylamide)
grafted cellulose nanocrystals as efficient flocculants for kaolin
suspension. Int. J. Biol. Macromol..

[25] Abitbol T., Marway H., Cranston E. D. (2014). Surface
modification of cellulose
nanocrystals with cetyltrimethylammonium bromide. Nordic Pulp Paper Res. J..

[26] Salajková M., Berglund L. A., Zhou Q. (2012). Hydrophobic
cellulose nanocrystals
modified with quaternary ammonium salts. J.
Mater. Chem..

[27] Nasseri R., Lee Y., Tam K. C. (2020). Interfacial
control of the synthesis of cellulose nanocrystal
gold nanoshells. Langmuir.

[28] Aguado R., Tarrés Q., Mutjé P., Pèlach M. À., Delgado-Aguilar M. (2022). Non-covalently
cationized nanocellulose
from hemp: Kinetics, key properties, and paper strengthening. Ind. Crops Products.

[29] Yin Y., Liu T., Wang B., Yin B., Yang Y., Russell T. P., Shi S. (2022). Nanoparticle/polyelectrolyte complexes for biomimetic constructs. Adv. Funct. Mater..

[30] Pomseethong P., Govindarasu M., Sharma G., Guo Y., Joy J. G., Kim S., Lee S. H., Kim J. C. (2026). Cellulose nanocrystal-based sulfatase-responsive
hydrogel for sustained celecoxib release in ulcerative colitis therapy. Biomater. Res..

[31] Wan W., Wang Y., Li Z., Wang X., Luo J. (2025). Polyethyleneimine
coated cellulose nanocrystals decorated with 3-carboxyphenylboronic
acid as a pH-responsive nanocarrier for targeted drug delivery. Cellulose.

[32] Ly M., Mekonnen T. H. (2020). Cationic surfactant modified cellulose nanocrystals
for corrosion protective nanocomposite surface coatings. J. Ind. Eng. Chem..

[33] Benkaddour A., Rusin C., Demir E. C., Ayranci C., McDermott M. (2023). Cationic surface
functionalization of cellulose nanocrystals and its effect on the
mechanical properties of polyamide 6 thin films. Cellulose.

[34] Derjaguin B. (1940). On the repulsive
forces between charged colloid particles and on the theory of slow
coagulation and stability of lyophobe sols. Trans. Faraday Soc..

[35] Verwey E. J. (1947). Theory
of the stability of lyophobic colloids. J. Phys.
Colloid Chem..

[36] Cao T., Elimelech M. (2021). Colloidal
stability of cellulose nanocrystals in aqueous
solutions containing monovalent, divalent, and trivalent inorganic
salts. J. Colloid Interface Sci..

[37] Pawcenis D., Lesniak M., Szumera M., Sitarz M., Profic-Paczkowska J. (2022). Effect of
hydrolysis time, pH and surfactant type on stability of hydrochloric
acid hydrolyzed nanocellulose. Int. J. Biol.
Macromol..

[38] Zhou Z., Xu J., Zhu S., Wang B., Li J., Ying G., Chen K. (2024). A gentle conditioning agent consisted of oppositely-charged-induced
cellulose nanocrystal and cationic cellulose: Stability, conditioning
and delivery. J. Cleaner Product..

[39] Zhang X. H., Goodwin Z. A. H., Hoane A. G., Deptula A., Markiewitz D. M., Molinari N., Zheng Q. L., Li H., McEldrew M., Kozinsky B., Bazant M. Z., Leal C., Atkin R., Gewirth A. A., Rutland M. W., Espinosa-Marzal R. M. (2024). Long-range
surface forces in salt-in-ionic liquids. ACS
Nano.

[40] Katana B., Takacs D., Szerlauth A., Saringer S., Varga G., Jamnik A., Bobbink F. D., Dyson P. J., Szilagyi I. (2021). Aggregation
of halloysite nanotubes in the presence of multivalent ions and ionic
liquids. Langmuir.

[41] Oncsik T., Desert A., Trefalt G., Borkovec M., Szilagyi I. (2016). Charging and
aggregation of latex particles in aqueous solutions of ionic liquids:
towards an extended Hofmeister series. Phys.
Chem. Chem. Phys..

[42] Mariño M. A., Rueda-Ordonez D., Paredes M. G., Tapia R. A., Pita R., Pavez P. (2024). Recycled ionic liquid vs. deep eutectic solvent in cellulose nanocrystals
production: Characterization, techno-economic analysis, and life cycle
assessment. J. Cleaner Product..

[43] Zhong W., Bobbink F. D., Fei Z., Dyson P. J. (2017). Polyimidazolium
salts: Robust catalysts for the cycloaddition of carbon dioxide into
carbonates in solvent-free conditions. ChemSusChem.

[44] Nečas D., Klapetek P. (2012). Gwyddion: an open-source
software for SPM data analysis. Open Phys..

[45] Grachev V., Deschaume O., Lang P. R., Lettinga M. P., Bartic C., Thielemans W. (2024). Dimensions
of cellulose nanocrystals from cotton and
bacterial cellulose: Comparison of microscopy and scattering techniques. Nanomaterials.

[46] Brinkmann A., Chen M. H., Couillard M., Jakubek Z. J., Leng T. Y., Johnston L. J. (2016). Correlating cellulose nanocrystal particle size and
surface area. Langmuir.

[47] Trefalt G., Szilagyi I., Oncsik T., Sadeghpour A., Borkovec M. (2013). Probing colloidal particle aggregation
by light scattering. Chimia.

[48] Szilagyi I., Rosicka D., Hierrezuelo J., Borkovec M. (2011). Charging and stability
of anionic latex particles in the presence of linear poly­(ethylene
imine). J. Colloid Interface Sci..

[49] Karateke B., Yılmaz N. D. (2025). Cellulose
nanocrystal extraction from okra and hemp
fibers. Cellulose.

[50] Boluk Y., Danumah C. (2014). Analysis of cellulose
nanocrystal rod lengths by dynamic
light scattering and electron microscopy. J.
Nanopart. Res..

[51] Grachev V., Lombardo S., Bartic C., Thielemans W. (2024). Thermodynamics
of interactions between cellulose nanocrystals and monovalent counterions. Carbohydr. Polym..

[52] Mikhailov V. I., Torlopov M. A., Martakov I. S., Krivoshapkin P. V. (2017). Stability
of nanocrystalline cellulose in aqueous KCl solutions. Colloid J..

[53] Brinatti C., Huang J., Berry R. M., Tam K. C., Loh W. (2016). Structural
and energetic studies on the interaction of cationic surfactants and
cellulose nanocrystals. Langmuir.

[54] Yeskie M. A., Harwell J. H. (1988). On the structure
of aggregates of adsorbed surfactants:
The surface charge density at the hemimicelle/admicelle transition. J. Phys. Chem..

[55] Alila S., Boufi S., Belgacem M. N., Beneventi D. (2005). Adsorption
of a cationic surfactant onto cellulosic fibers I. Surface charge
effects. Langmuir.

[56] Su X., Wan Z., Lu Y., Rojas O. J. (2024). Control of the colloidal and adsorption
behaviors of chitin nanocrystals and an oppositely charged surfactant
at solid, liquid, and gas interfaces. Langmuir.

[57] Hierrezuelo J., Sadeghpour A., Szilagyi I., Vaccaro A., Borkovec M. (2010). Electrostatic
stabilization of charged colloidal particles with adsorbed polyelectrolytes
of opposite charge. Langmuir.

[58] Asserzhanov D. K., Grachev V., Hornok V., Klivenko A. N., Abdullina E. S., Kassymova Z. S., Szilagyi I. (2026). Electrosteric forces drive the stability
and antibacterial function of polysaccharide-stabilized silver nanoparticles. J. Mol. Liq..

[59] Trefalt G., Szilagyi I., Borkovec M. (2020). Schulze-Hardy rule revisited. Colloid Polym. Sci..

[60] Takács D., Adzic M., Omerovic N., Vranes M., Katona J., Pavlovic M. (2024). Electrolyte-induced aggregation of
zein protein nanoparticles
in aqueous dispersions. J. Colloid Interface
Sci..

[61] Shen H., Wang T., Xu X. (2026). Construction and performance of polyethyleneimine
modification on cellulose nanocrystal surface and reducing end groups. Int. J. Biol. Macromol..

[62] Khandal D., Riedl B., Tavares J. R., Carreau P. J., Heuzey M.-C. (2019). Tailoring
cellulose nanocrystals rheological behavior in aqueous suspensions
through surface functionalization with polyethyleneimine. Phys. Fluids.

[63] Wei W. C. (2023). Hofmeister
effects shine in nanoscience. Adv. Sci..

[64] Manning G. S. (1969). Limiting
laws and counterion condensation in polyelectrolyte solutions 1. Colligative
properties. J. Chem. Phys..

