# Supplementary material for: Cationization of Cellulose Nanocrystals with Ionic Liquids and Polymers: From Electrostatic Screening to Adsorption-Controlled Aggregation
Source: Langmuir. 2026 Aug 7;42(32):23471–80. doi: 10.1021/acs.langmuir.6c02450 (PMC13492382; doi:10.1021/acs.langmuir.6c02450)
Supplement: Supplementary file 1 [file la6c02450_si_001.pdf]

TABLE OF CONTENT

Title page S2

Figure S1. AFM image and size distributions of bare sCNCs S3

Figure S2. AFM images of polymer-coated sCNCs S4

Figure S3. Hydrodynamic radius distributions S5

Figure S4. Stability ratio and zeta potential in salts S6

Figure S5. Photograph of bPEI-coated sCNCs S7

Table S1. Elemental analysis S8

Table S2. CCC values for ionic liquids and surfactants S9

Table S3. AFM dimensions S10

Table S4. CCC values in monovalent salts S11

Table S5. CCC values in multivalent salts S12

Supporting Information (SI)

Cationization of Cellulose Nanocrystals with Ionic Liquids and Polymers: From Electrostatic Screening to Adsorption Controlled Aggregation

Vladimir Grachev[‡]*, *Viktória Hornok,*[‡] *Wim Thielemans*[§] *and István Szilágyi*[‡]*

[‡]MTA-SZTE "Lendület" Biocolloids Research Group, Department of Physical Chemistry and Materials Science, Interdisciplinary Centre of Excellence, University of Szeged, 6720 Szeged, Hungary

[§]Sustainable Materials Lab, Department of Chemical Engineering, KU Leuven, Campus Kulak Kortrijk, 8500 Kortrijk, Belgium

*Corresponding authors. Email: grachev.vladimir@szte.hu (V.G.), szistvan@chem.u-szeged.hu (I.S.)

Number of pages: 11

Number of figures: 5

Number of schemes: 0

Number of tables: 5

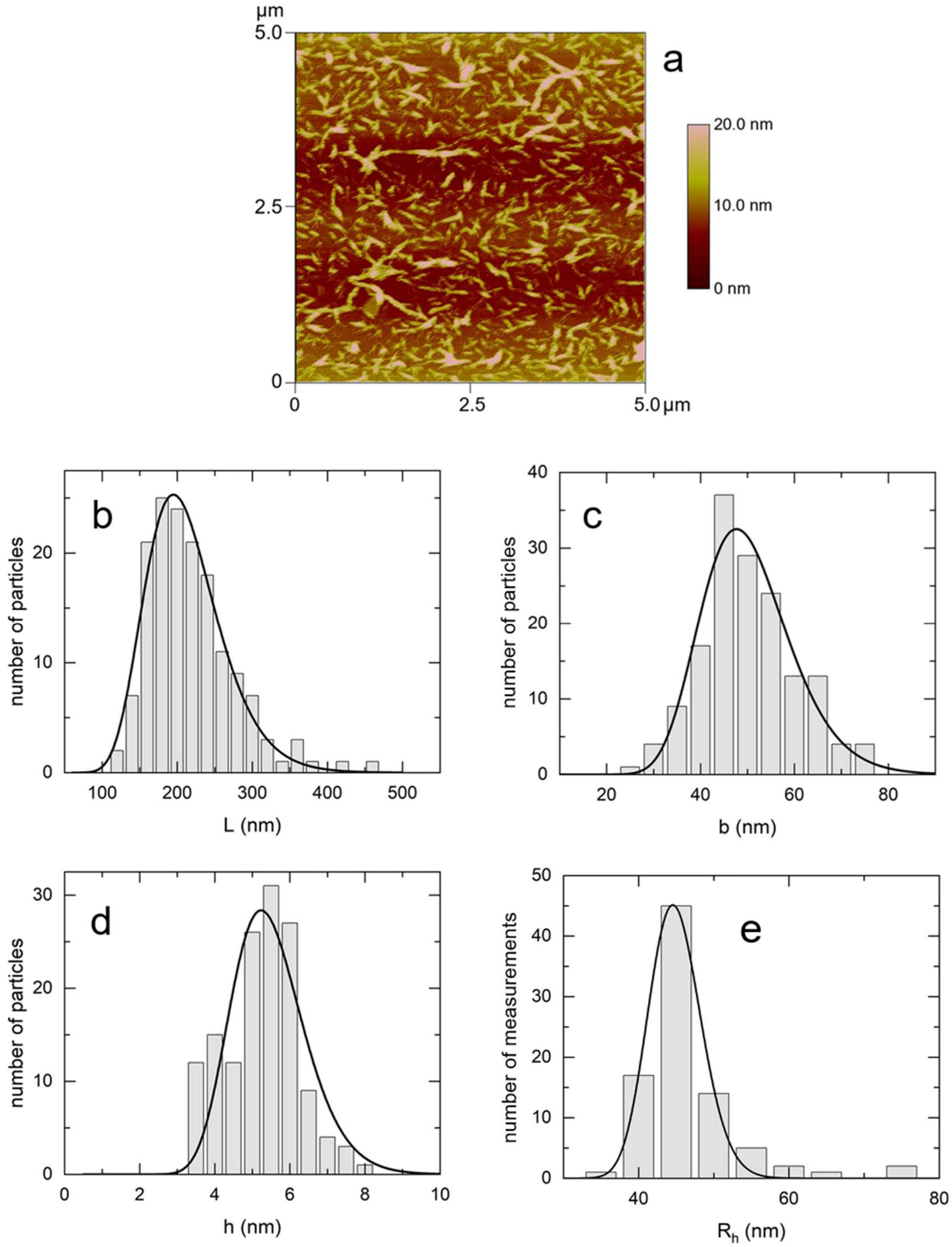

Figure S1. AFM image (a) and distributions in length (b), width (c), height (d) and hydrodynamic radius (e) for bare sulfated cellulose nanocrystals (sCNCs).

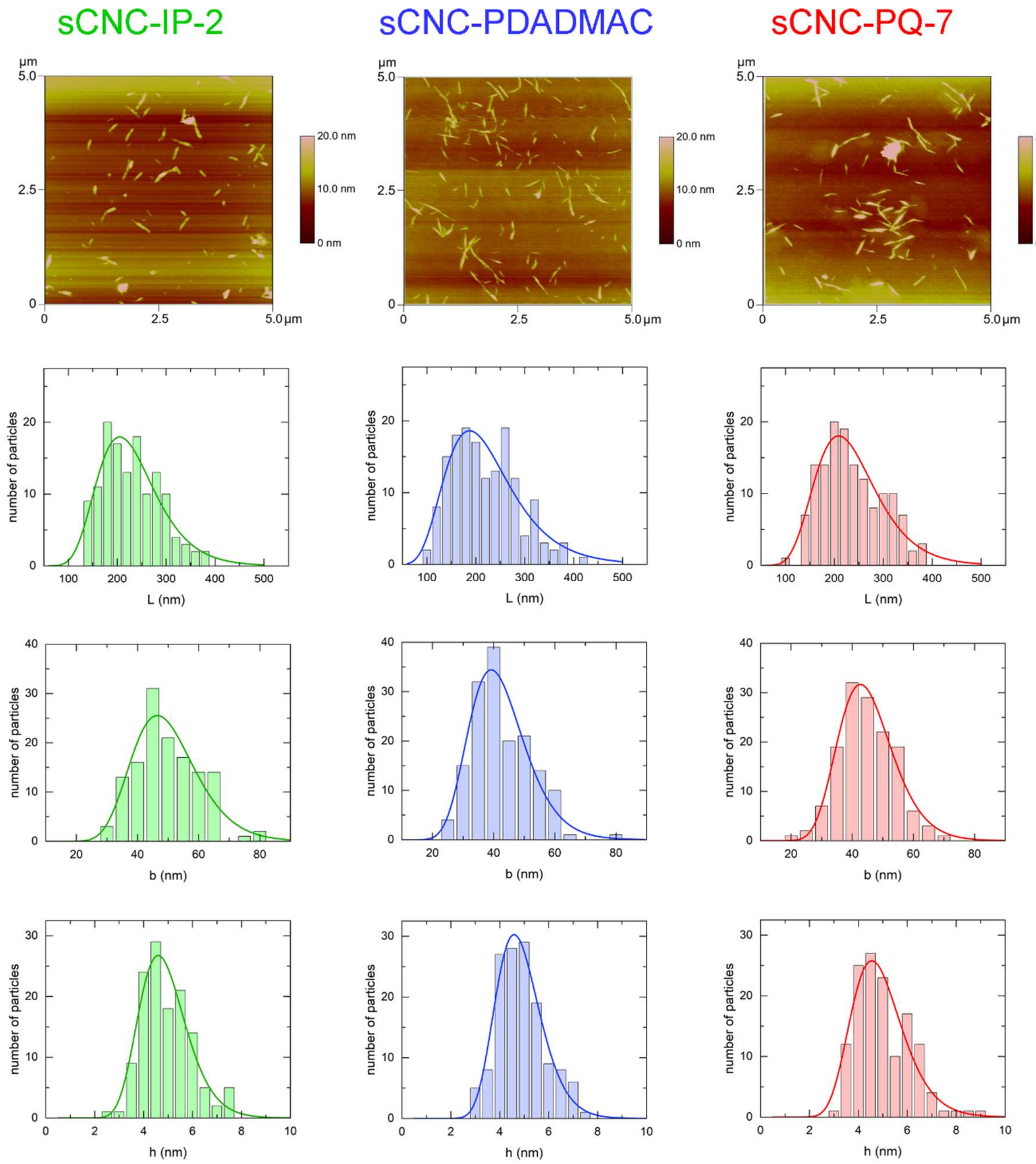

Figure S2. AFM images and distributions in length (L), width (b) and height (h) for polymer-coated sCNCs.

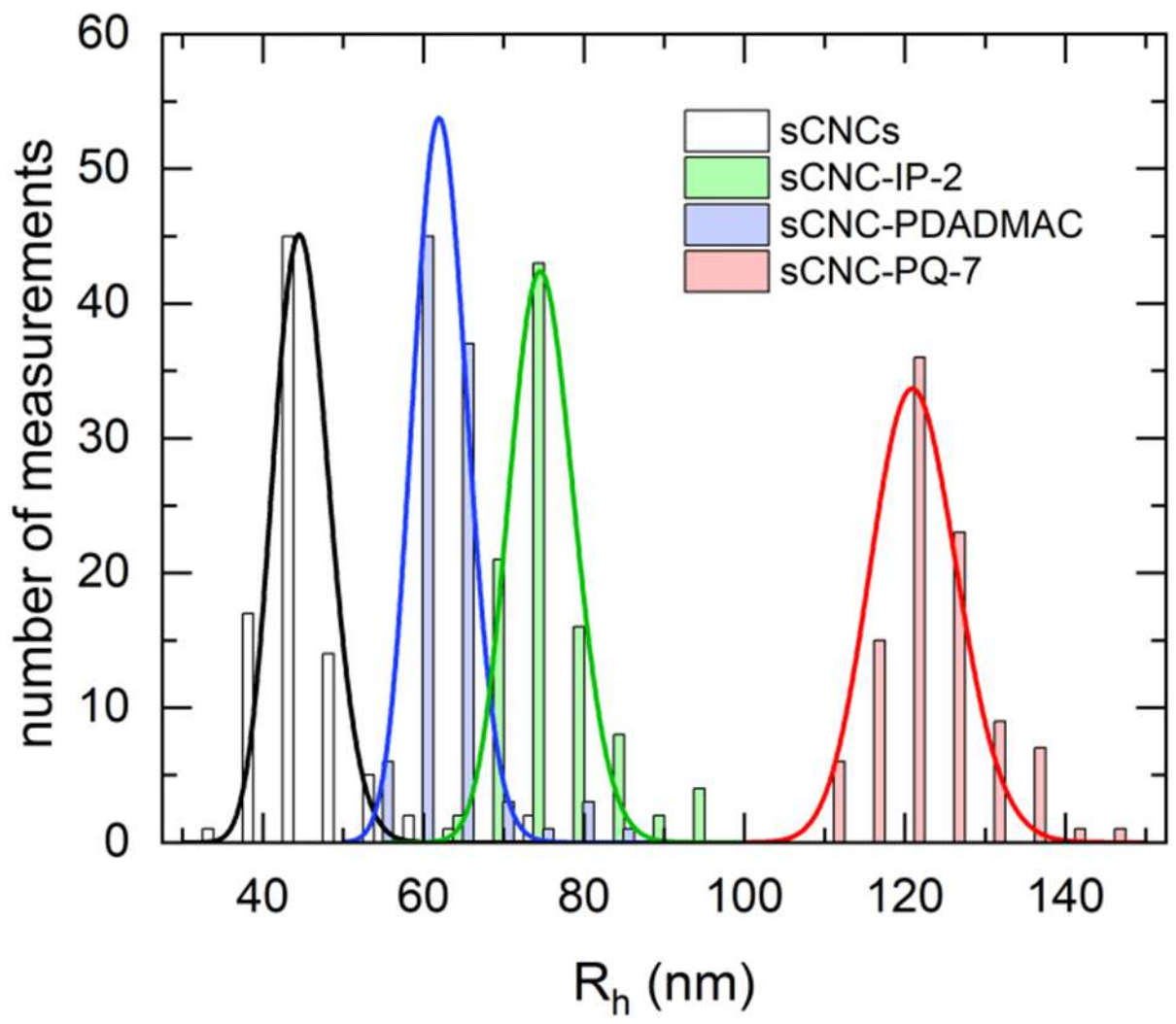

Figure S3. Hydrodynamic radius distributions of bare and polymer-coated sCNCs.

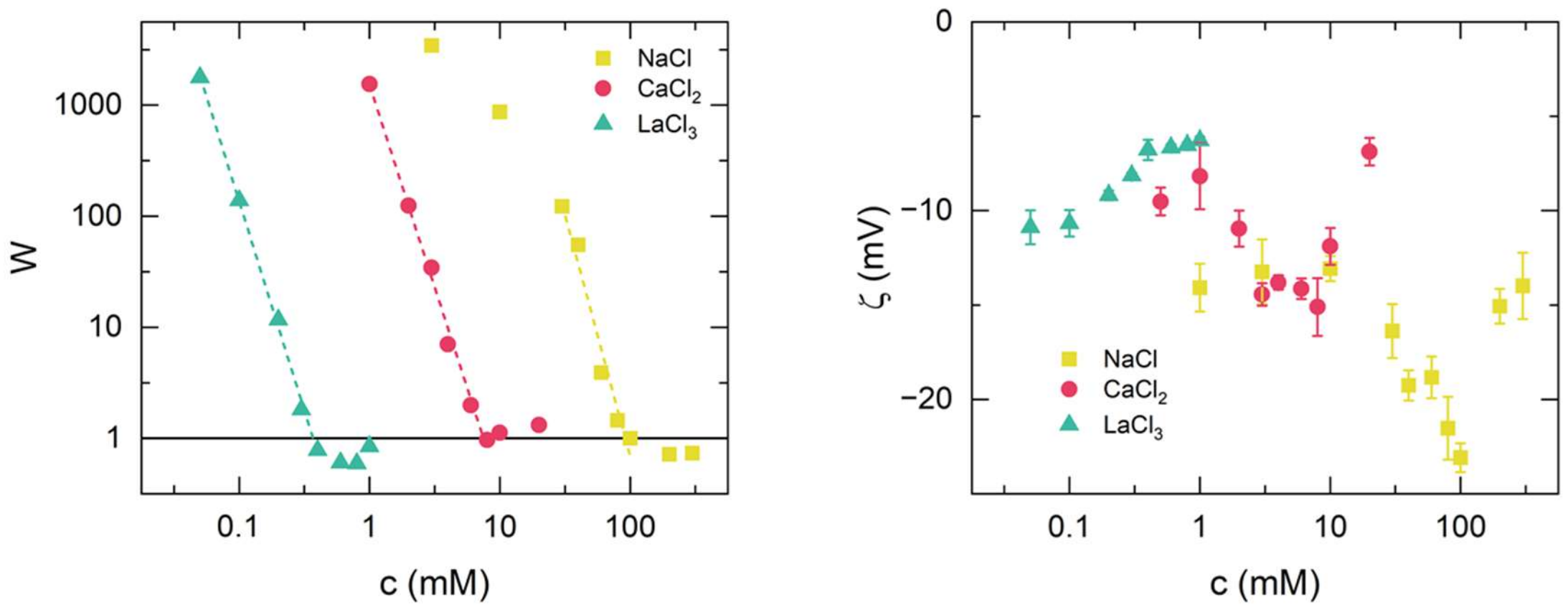

Figure S4. Stability ratio (W) and zeta potential (ζ) data of bare sCNC particles as a function of the concentration of different salts.

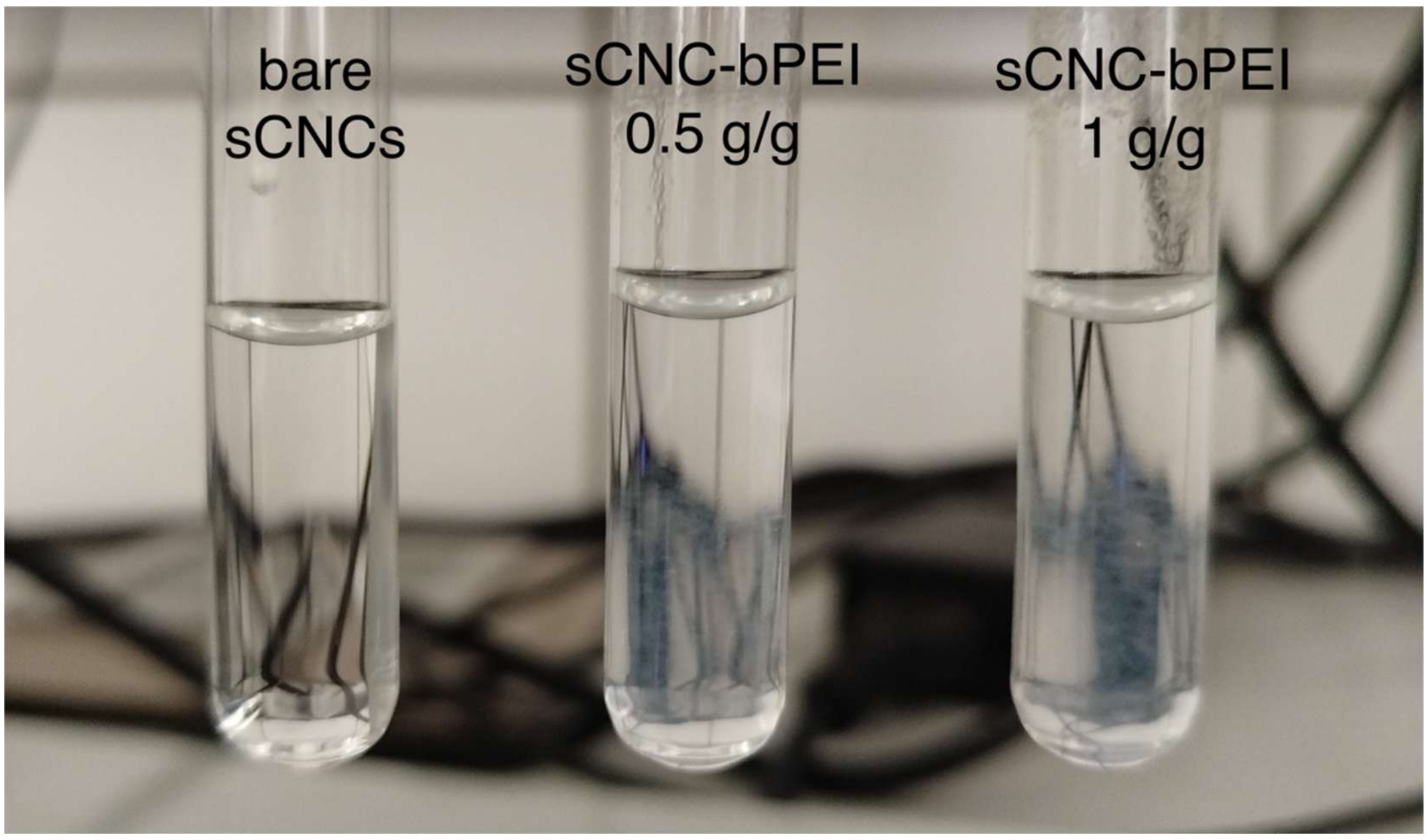

Figure S5. Photograph of bare and bPEI-coated sCNCs at a particle concentration of 100 mg/L.

Table S1. Mass fractions of carbon, hydrogen, nitrogen and sulfur in bare sCNCs as determined by elemental analysis.

**element**	**mass fraction (mg/g sCNCs)**
C	405.88±2.14
H	60.64±0.38
N	1.63±0.03
S	5.96±0.12

Table S2. Critical coagulation concentration (CCC) data of sCNCs in the presence of alkylimidazolium ILs of different N_C values, NaCl and cationic surfactants. The critical micelle concentrations (cmc) and isoelectric points (IEP) are indicated wherever available.

compound	N_C	CCC (mM)	IEP (mM)	cmc	CCC-to-cmc ratio
mimCl	0	81.8±11.9	-	-	-
emimCl	2	102.1±10.7	-	-	-
bmimCl	4	98.5±12.6	-	-	-
hmimCl	6	93.2±18.9	-	-	-
omimCl	8	73.7±13.6	83.3±9.4	227[1]	0.32±0.06
dmimCl	10	14.0±4.6	28.8±4.5	56.8[1]	0.25±0.08
NaCl	-	85.1±15.3	-	-	-
DTAB	-	3.1±0.1	4.3±0.6	14[2]	0.22±0.01
CTAB	-	0.12±0.02	0.12±0.03	0.8[2]	0.15±0.03

Table S3. Mean dimensions (length $\langle L \rangle$, width $\langle b \rangle$ and height $\langle h \rangle$) and their respective polydispersities (polydispersity in length σ_L, in width σ_b and in height σ_h) of polymer-coated sCNCs compared to bare sCNCs.

parameter	bare sCNCs	sCNC-IP-2	sCNC-PDADMAC	sCNC-PQ-7
$\langle L \rangle$ (nm)	212±52	232±67	225±82	238±71
σ_L	0.24±0.01	0.28±0.02	0.35±0.03	0.29±0.02
$\langle b \rangle$ (nm)	50±10	50±11	42±10	45±9
σ_b	0.19±0.01	0.22±0.02	0.23±0.02	0.20±0.01
$\langle h \rangle$ (nm)	5.5±1.0	4.9±1.0	4.9±1.0	4.9±1.2
σ_h	0.18±0.02	0.21±0.01	0.20±0.01	0.22±0.02

Table S4. Critical coagulation concentration (CCC) of polymer-coated sCNCs in the presence of monovalent salts.

salt	CCC (mM)		
	sCNC-IP-2	sCNC-PDADMAC	sCNC-PQ-7
NaCl	5.3±1.2	83±17	39±12
NaBr	5.8±1.3	47±11	19±7
NaI	4.3±1.6	31±13	16±11
CH_3COONa	5.4±1.9	501±31	71±9
$NaHCO_3$	4.6±1.2	687±67	23±3

Table S5. Critical coagulation concentration (CCC) data for bare and polymer-coated CNCs in the presence of multivalent salts.

**salt**	**CCC (mM)**			
	bare sCNCs	sCNC-IP-2	sCNC-PDADMAC	sCNC-PQ-7
NaCl	92±18	5.3±1.2	83±17	39±12
Na_2SO_4	7.5±0.5	0.34±0.08	3.5±0.6	1.5±0.4
Na_3cit	0.37±0.02	0.02±0.01	0.07±0.01	0.14±0.02

REFERENCES

(1) Jungnickel, C.; Łuczak, J.; Ranke, J.; Fernández, J. F.; Müller, A.; Thöming, J. Micelle formation of imidazolium ionic liquids in aqueous solution. *Coll. Surf. A* **2008**, *316*, 278-284.

(2) Bahri, M. A.; Hoebeke, M.; Grammenos, A.; Delanaye, L.; Vandewalle, N.; Seret, A. Investigation of SDS, DTAB and CTAB micelle microviscosities by electron spin resonance. *Coll. Surf. A* **2006**, *290*, 206-212.
